# On Syphilis, Constitutional, and Hereditary: And on Syphilitic Eruptions

**Published:** 1853-06

**Authors:** 


Art. VI.—*On Syphilis, Constitutional and Hereditary; and on Syphilitic Eruptions.* By Erasmus Wilson, F. R. S. Philadelphia: Blanchard & Lea. 1852. 8vo pp. 284.

The author of this work is well known to American readers by his System of Human Anatomy, and a treatise on diseases of the skin. The popularity of these works, especially of the former, has been very great, and that will probably lead to a wide circulation of the volume before us. Mr. Wilson is not a mere compiler on the subject of syphilis, but an original observer and thinker, and on some points entertains views peculiar to himself. He carries the humoral pathology further than any writer whom we have lately read. For example, he holds that the system of a man or woman may become so saturated with the virus of syphilis as to be able to communicate the disease, by the secretions of the body, to a healthy person; and in proof of the position cites the following example:

"A gentleman had a small venereal sore on the prepuce in the month of November; it got well speedily. In the succeeding month of February he suffered from sore-throat and rheumatic pains, but of so slight a nature that he took them to be merely symptoms of a common cold. In March he married.

"In the month of June following, I was called to see his wife, in consultation with her medical man. She was suffering a furuncular abscess, situated at the upper and inner part of the thigh, and my opinion was sought more on account of the obstinate nature of the sore which the abscess had left behind it, than from any suspicion of its being of a venereal nature. I was struck by the unhealthy-looking, red, and fungous surface which the sore exhibited; and making further i iquiries found that on the day before she had perceived a rash upon her skin which had now become an unmistakeable roseola. She had a feeling of soreness in the throat, but without congestion, and her skin was muddy and discolored.

"She had no disorder of the genital organs, and her husband was free from any symptoms of disease.

"I apprehend that, in this case, no one can doubt the natural secretions of the mucous membrane of the husband being the medium of transmission of the poison; and as there was no local disease in the wife, the poison must have been imbibed into her system at once, and thus have contaminated her blood. Then came the period of latency, during which the poison was accumulating and gaining strength, and then followed the outburst of exanthematic eruption. The furuncular abscess I consider to have been of a common kind (at the time there existed a kind of furuncular epidemic) in the first instance; its angry appearance and its indisposition to heal having been occasioned subsequently by the approach of the syphilitic crisis."

Mr. Wilson contends that in this affection all the secretions become poisoned, and that in this way infants tainted with it may communicate syphilis to the nurse. We cannot help believing that some of these points he has strained too far, going beyond what observation and experiment sanction. Children, no doubt, do often affect the mother or nurse, but it is hardly through the saliva. It will be found, in such cases, we apprehend, that the child has syphilitic sores about the mouth.

The following is Mr. Wilson's theory of the action of the syphilitic poison:

"When this poison is once admitted into the human organism it has a tendency to accumulate until it attains a certain point, which may be termed the *point of saturation*. As soon as the saturating point is reached, an outburst of fever, which results in the elimination of the excess of collected poison, takes place, and the system returns to its wonted tranquility and calm. This process is repeated at intervals, until after a time the intervals lengthen, and the effects gradually diminish; from weeks, the intervals become months, and years; from severe fever, the attacks become trivial and insignificant; and at last the poison is so thoroughly assimilated, that it ceases to accumulate in excessive quantities, and loses its power of exciting a febrile action in the blood of the infected person.

"But although it may be incapable of exciting disease among tissues accustomed to its presence, it still retains the power of contaminating new blood; and it is difficult to determine how long this degree of virulence continues. At first, probably, it may be so far weakened that the wife escapes, but the offspring may suffer; and at last it is rendered so mild, that only accidental conditions call up its powers of doing evil. It remains, however, as I believe, lurking in the blood and in the tissues for many years, and probably for the rest of life.

"Under these circumstances, our answer to the question as to the time which should intervene between disease and marriage, must necessarily be modified by a variety of conditions; for example, by the nature of the secondary disease, by the known susceptibility of the individual, by his state of health, his occupation, and by the treatment he may have undergone; and something must be known also of the health of the proposed wife. Taking the most favorable view of the case, from two to five years should be permitted to elapse, such period being passed under the close observation of the medical man."

Our author is an advocate for the doctrine of secondary gonorrhœa. He says: "When we see a man perfectly free from any primary symptoms of disease, communicating syphilis to his newly-married wife, by his secretions alone, can we doubt the possibility of a similar result accruing from a syphilitic secretion poured out by the mucous membrane, as happens in gonorrhœa" It is only a few cases of gonorrhœa, that, in his opinion, can generate syphilis, but those few, he contends, "have as much the power of transmitting" that disorder "as an undoubted chancre." The following case he gives in illustration:

"In 1828, a medical man had gonorrhœa, but neither excoriation nor sore. In the following year, after getting wet through, he was attacked with rheumatic fever, eruption on the skin, and iritis. Ten years afterwards he again suffered from gonorrhœa.

"In 1840, he was annoyed with nocturnal pains in the tibiæ; for these he took vapor baths, which brought out an eruption on the skin; and with the latter he has been troubled from that time until the present.

"In the month of May, 1850, he consulted me for an eruption on the face of small, soft, syphilitic tubercles.

One of the tubercles was situated on the upper eyelid, two or three occupied the ala of the nose on one side; a small cluster were collected on the lower jaw amid the hair of the whisker, and there was one on the hard palate.

"I select this case as being that of a man well able to judge of his own symptoms, and to form a clear idea of the nature of his ailment. He told me that he felt convinced that he could not have had a chancre in the urethra."

The physician "felt convinced that he could not have had a chancre in the urethra." But how could he be sure of it? It was his belief; but shall we accept this conviction of the patient against the uniform observation of the medical world? Mr. Wilson, we must believe, was in haste to adopt this conclusion. Upon most points he coincides with Ricord, to whom he dedicates his book, and whose investigations of syphilis have exceeded in patience, variety, duration, accuracy and extent those of any other author of modern or ancient times. We conclude our brief notice of his interesting work by an extract from the chapter on "Secondary and Constitutional Syphilis."

"After the lapse of a short but variable period, namely, from one week to three or more, the local or primary disease becomes exhausted, the sore or ulcer heals, and the patient is to all appearance well.

"It may be so, in fact; the poison may have gone no farther than the part which has been ravaged by the disease, it may have become exhausted on the spot, or it may have been transmitted through the bloodvessels into the stream of life, have poisoned the entire mass of blood, and, by means of the blood, all the tissues of the body. It may be there *latent*, but not lost, silently accumulating, and ready on a sudden to burst forth in a new shape, namely, as a general or constitutional disease, as, in fact, a syphilitic fever.

"It is a well known law of animal poisons, that, being once introduced into the blood, they excite in that fluid an action which has for its object the production of a similar poison, and this process goes on until the blood becomes saturated or overcharged with the morbific principle. As soon as this latter condition occurs, an inflammatory movement is set up, which results in the ejection or elimination of the poison.

"This inflammatory movement, or syphilitic fever, is therefore a sign of the accumulation of the poison within the blood, to such a degree as to disturb the healthy functions of the body, and is attended with symptoms which indicate derangement of the nervous, vascular and digestive systems, and especially of those surfaces of the body through which it is possible for elimination to occur.

"The blood, it must be recollected, is charged with a poisonous principle, and it may therefore be concluded that all the organs and structures supplied with that blood must suffer to a greater or less extent. The brain evinces its suffering by mental dejection; the nerves, by a general feeling of prostration and debility. Everything is *couleur de plomb* around the patient; he is unable to pursue his avocations with comfort, and if they require the exercise of his mind, scarcely at all. He is oppressed with a sense of impending evil. Besides the lassitude and languor which evince the poisoned condition of the nerves, there is often neuralgia to an intense degree, sometimes affecting the head or face, and sometimes the joints, when it goes under the name of rheumatism. The neuralgia presents the peculiarity of being nocturnal, that is, of being most severe during the night, and often, but not always, entirely absent by day. The pulse is quickened; the tongue is coated, white, broad, and indented by the teeth. The fauces are more or less congested, the tonsils and soft palate being frequently swollen; there is irritation of the larynx, producing a mucous cough, and often nausea. The bowels are sometimes constipated, sometimes relaxed; the urine sometimes clear and limpid, at other times loaded with salts. The conjunctiva is congested and muddy, and the whole skin remarkable for its yellowish and dirty appearance, looking as if saturated with impure and discolored humors. Sometimes it is dry; at others suffused with a greasy secretion, and at night pours out an abundant and fetid perspiration.

"Such are the general symptoms of the syphilitic fever, or secondary syphilis, but they may not all be present, and those which exist may be complicated by local congestions of the mucous membranes. The symptoms which may be selected as pathognomonic of syphilitic fever are: mental and nervous depression and prostration; congested fauces, with sore-throat; congested and muddy conjunctiva; congested and discolored skin, the congestion being partial or general, and assuming the form of an eruption; and, added to these, neuralgic pains.

In this combination of symptoms we are forcibly struck with the resemblance which they bear to those of the exanthematous fevers, measles, scarlatina, and smallpox. Firstly, the nervous depression, showing the stagnating influence of the accumulated poison. Secondly the congestion of the mucous membranes, particularly of the fauces, showing the effort made by the bloodvessels to eject the poison through that tissue. And thirdly, the cutaneous exanthema, which completes the triumph of the pressure from within, and is the sign that the poison is driven to the surface and is in process of expulsion.

"Even the irregular symptoms, the partial and local congestions, have their parallel among the exanthemata.

"Thus far for resemblances to the exanthematic fevers, but there are also differences between the syphilitic fever and that of the exanthemata, so remarkable as to call for special consideration. The exanthematous fevers are more violent, more regular, and more transient than the syphilitic fever, in other words, they are *acute*, while the syphilitic fever is *chronic*. It is true that instances of syphilitic fever often happen, which present all the symptoms of the most violent fever, and are attended with delirium; but such cases are the exception and not the rule.

"The cause of the differences of character perceptible between the exanthematous and the syphilitic fever appears to me to be due to a radical difference in the nature of the poison. The poison of measles, scarlatina, and smallpox probably originates in conditions extraneous to the animal body; it reaches the blood as an element foreign to its nature, and as soon as it has accumulated to the saturating point, a violent effort is made for its expulsion. The expulsive effort obeys rigidly certain laws of order and time, and the poison being once removed, the blood of the patient enjoys an immunity from a re-excitement of the same action for the rest of life."

